# Supplementary material for: A Type VI Secretion System Trans-Kingdom Effector Is Required for the Delivery of a Novel Antibacterial Toxin in Pseudomonas aeruginosa
Source: Front Microbiol. 2019 Jun 5;10:1218. doi: 10.3389/fmicb.2019.01218 (PMC6560169; doi:10.3389/fmicb.2019.01218)
Supplement: Supplementary file 1 [file Data_Sheet_1.PDF]

AT6SS trans-kingdom effector is required for the delivery of a novel antibacterial toxin in *Pseudomonas aeruginosa*

Benjamin Berni[1], Chantal Soscia[1], Djermoun Sarah[1], Ize Bérengère[1] and Sophie Bleves[1]*

Supplementary data

Supplementary figure 1: *In silico* analysis of the nucleic and protein sequence of Tla3 (PA0259).

Supplementary figure 2: Production of Tle3^{p} (S2A) and localization of Tle3^{c}, Til3 and Tla3$_{ATG1}$ in *E. coli* BL21(DE3)pLysS (S2B-D).

Supplementary figure 3: Bacterial two-hybrid assay.

Supplementary figure 4: *P. aeruginosa* growth competition.

Supplementary figure 5: Adaptors of Tla3 family DUF2875 are restricted to beta and gamma proteobacteria.

Supplementary figure 6: Original blots from figures 4 and 5

Table 1: *E. coli* and *P. aeruginosa* strains and plasmids used in this study.

	Genotype or description	Origin
**Strains**		
E. coli		
DH5α	F-, Δ(*argF -lac*)U169, *phoA* , *supE44* , Δ(lacZ)M15, *relA* , *endA* , *thi* , *hsdR*	Invitrogen
CC118λpir	Δ(*ara-leu*) *ara* D Δ*lac* X74 *gal* E *gal* K *pho* A20 *thi* -1 *rps* E *rpo* B *arg* E (Am) *rec* A1 RfR (λpir)	Laboratory collection
BL21(DE3)PlysS	F– *ompT gal dcm lon hsdSB(rB–mB–*) λ(DE3 [*lacI lacUV5-T7p07 ind1 sam7 nin5*]) [*malB+]K-1* 2(λS) pLysS[T7p20 orip15A, CmR	Laboratory collection
BTH101	F-, *cya-99, araD139, galE15, galK16, rpsL1 (Str r), hsdR2, mcrA1, mcrB1.*	Laboratory collection
P. aeruginosa		
PAO1	Wild-type prototroph, cytotoxic and invasive	Laboratory collection
PAO1 *tla3* V5	chromosomally encoded Tla3V5 translational fusion in PAO1	This work
PAO1 △*rsmA*	*rsmA* deletion mutant	This work
PAO1 *hcp2b 6His*	chromosomally encoded H*cp2b 6His* translational fusion in PAO1	This work
PAO1 △*rsmA hcp2b His*	chromosomally encoded *hcpC6His* translational fusion in PAO1 △*rsmA*	This work
PAO1 △*rsmA* △*clpV2 hcp2b His*	chromosomally encoded *hcpC6His* translational fusion in PAO1 △*rsmA* △*clpV2*	This work
PAO1 △*rsmA* △*tle3 hcp2b His*	chromosomally encoded *hcpC6His* translational fusion in PAO1 △*rsmA* △*tle3*	This work
PAO1 △*rsmA* △*tla3 hcp2b His*	chromosomally encoded *hcpC6His* translational fusion in PAO1 △*rsmA* △*tla3 (PA0259)*	This work
PAO1 tli3V5	chromosomally encoded Tli3V5(PA0261) translational fusion in PAO1	This work
PAO1 *tla3V5*	chromosomally encoded Tla3V5(PA0259) translational fusion in PAO1	This work
PAO1△*clpV2*	*clpV2* deletion mutant	Sana et al., 2012
PAO1△*vgrG2b*	*vgrG2b* deletion mutant	Sana et al., 2015
PAO1△*tli3* △*tle3*	*tli3 (PA0261)* and *tle3* deletion mutant	This work
PAO1△*tle3*	*tle3* deletion mutant	This work
PAO1△*tla3*	*tla3 (PA0259)* deletion mutant	This work
PAO1△*tli3* △*tle3 attB :: tli3+*	*cis* complementation of △*tli3* △tle3 mutation by a *tli3* copy inserted at the *attB* site on *P. aeruginos* a chromosome	This work
PAO1△*tle3* attB :: *tle3* +	*cis* complementation of △*tle3* mutation by a *tle3* copy inserted at the *attB* site on *P. aeruginosa* chromosome	This work
PAO1△*tla3* attB :: *PA0259* +	*cis* complementation of △*tla3* mutation by a *tla3* copy inserted at the *attB* site on *P. aeruginosa* chromosome	This work
**Plasmids**		
pRK2013	ColE1 origin, Tra+, mob+, KmR	Laboratory collection
pKNG101	R6K origin, TRK2 origin, mobRK2, sacBR+, SmR	Kaniga et al., 1991
pKNG101△*rsmA*	suicide vector for *rsmA* deletion, ,SmR	Mercy et al., 2016
pBB29	suicide vector for *tle3* deletion, SmR	This work
pBB30	suicide vector for *tla3* (PA0259) deletion, SmR	This work
pBB31	suicide vector for *tli3 (PA0261)* and *tle3* deletion, SmR	This work
pBB32	suicide vector for *tli3* V5 insertion, SmR	This work
pBB33	suicide vector for *tla3* V5 insertion, SmR	This work
pBB34	suicide vector for *hcp2b* 6His insertion, SmR	This work
pBB35	suicide vector for ATG1 substitution in ATA upstream the annotated ATG of *tla3* (*PA0259)* , SmR	This work
pBB36	suicide vector for ATG3 substitution in ATA upstream the annotated ATG of t*la3 (PA0259)* , SmR	This work
pBB37	suicide vector for annotated ATG substitution in ATA of *tla3 (PA0259)* , SmR	This work
Mini-CTX1	Contains *attP* site for integration at the *attB* site of *P. aerugionosa* chromosome, TcR	Hoang et al., 2000
pBB38	*tle3* under control of *hcp2b* promoter cloned in EcoRI of Mini-CTX1, TcR	This work
pBB39	*tli3 (PA0261)* under control of *hcp2b* promoter cloned in EcoRI of Mini-CTX1, TcR	This work
pBB40	*tla3 (PA0259)* under control of *hcp2b* promoter cloned in EcoRI of Mini-CTX1, TcR	This work
pJN105	*P. aeruginosa* expression vector with an arabinose inducible pBAD promoter, GmR	Newman et al., 1999
pUT18	Bacterial Two Hybrid vector, ColE1 origin, P*lac* , T18 fragment of *Bordetella pertussis* CyaA, AmpR	Euromedex
pBB1	*tle3* cloned downstream the T18 coding sequence in pUT18	This study
pBB2	*tle3(4-380)* cloned downstream the T18 coding sequence in pUT18	This study
pBB3	*tle3(384-717)* cloned dowstream the T18 coding sequence in pUT18	This study
pBB4	*PA0261* cloned downstream the T18 coding sequence in pUT18	This study
pBB5	*PA0259* cloned downstream the T18 coding sequence in pUT18	This study
pBB6	*PA0259(21bp upstream ATG3-244)* cloned downstream the T18 coding sequence in pUT18	This study
pBB7	*PA0259(247-381)* cloned downstream the T18 coding sequence in pUT18	This study
pBB8	*PA0262* (1-2) cloned downstream the T18 coding sequence in pUT18	This study
pBB9	*PA0262* (1-2-3) cloned downstream the T18 coding sequence in pUT18	This study
T18-Pal	*pal* cloned downstream the T18 coding sequence in pUT18	Battesti et al., 2012
pKNT25	Bacterial Two Hybrid vector, p15A origin, P*lac* , T25 fragment of *Bordetella pertussis* CyaA, KanR	Euromedex
pBB10	*tle3* cloned downstream the T25 coding sequence in pkNT25	This study
pBB11	*PA0261* cloned downstream the T25 coding sequence in pkNT25	This study
pBB12	*PA0259* cloned downstream the T25 coding sequence in pkNT25	This study
pBB13	*PA0262* (1-2) cloned downstream the T25 coding sequence in pkNT25	This study
pBB14	*PA0262* (1-2-3) cloned downstream the T25 coding sequence in pkNT25	This study
pUT18C	Bacterial Two Hybrid vector, ColE1 origin, P*lac* , T18 fragment of *Bordetella pertussis* CyaA, AmpR	Euromedex
pBB15	*tle3* cloned upstream the T18 coding sequence in pUT18C	This study
pBB16	*PA0261* cloned upstream the T18 coding sequence in pUT18C	This study
pBB17	*PA0259* cloned upstream the T18 coding sequence in pUT18C	This study
pBB18	*PA0262* (1-2) cloned usptream the T18 coding sequence in pUT18C	This study
pBB19	*PA0262(1-2-3)* cloned upsteam the T18 coding sequence in pUT18C	This study
pKT25	Bacterial Two Hybrid vector, p15A origin, P*lac* , T25 fragment of *Bordetella pertussis* CyaA, KanR	Euromedex
pBB20	*tle3* cloned upstream the T25 coding sequence in pKT25	This study
pBB21	*PA0261* cloned upstream the T25 coding sequence in pKT25	This study
pBB22	*PA0259* cloned upstream the T25 coding sequence in pKT25	This study
pBB23	*PA0262* (1-2) cloned usptream the T25 coding sequence in pKT25	This study
pBB24	*PA0262(1-2-3)* cloned upsteam the T25 coding sequence in pKT25	This study
ToLB-T25	*tolB* cloned upstream the T25 coding sequence in pkT25	Battesti et al., 2012
pRSFDUET-1	Expression vector, RSF origin, *lacI* , PT7, KanR	Novagen
pBB25	*vgrG2b* cloned into pRSFDUET-1, C-terminal Strep epitope, KanR	This study
pBB26	*PA0261* downstream the signal sequence cloned into pRSFDUET-1, C-terminal Strep epitope, KanR	This study
pVT8	*PA0261* cloned into pRSFDUET-1, C-terminal V5 epitope, KanR	This study
pBB27	*PA0259* from ATG3 cloned into pRSFDUET-1, C-terminal Strep epitope, KanR	This study
pVT9	*PA0259* from ATG1 cloned into pRSFDUET-1, C-terminal V5 epitope, KanR	This study
pSBC107	insert from pSBC81 cloned into pVT8 leading to *tli3* and the sequence coding Tle3P on the same transcript, KanR	This study
pSBC110	insert from pBB44 cloned into pVT8 leading to *tli3* and the sequence coding CterVgrG2bP on the same transcript, KanR	This study
pETDuet-1	Expression vector, ColE1 origin, *lacI* , *PT7* , ApR	Novagen
pVT1	*tle3* cloned into pETDUET-1, C-terminal 10His epitope, ApR	This study
pBB28	*tla3 (PA0259)* from *ATG3* cloned into pETDUET-1, C-terminal 10His epitope, ApR	This study
pET22b	Expression vector, PBR322 origin, PT7, PelB signal sequence, ApR	Invitrogen
pSBC81	*tle3* cloned downstream the PelB signal sequence in pET22b, C-terminal 10His epitope, ApR	This study
pVT3	*tle4* cloned into pETDUET-1, C-terminal 10His epitope, ApR	This study
pBB41	*PA0262* (△3) cloned into pRSF-DUET, C-terminal Strep epitope, KanR	This study
pBB42	*PA0262* (△2-3) cloned into pRSF-DUET, C-terminal Strep epitope, KanR	This study
pBB43	*PA0262* (1-2-3) cloned into PCDF-DUET, C-terminal 10His epitope,	This study
pBB44	*PA0262* (1-2-3) cloned downstream the PelB signal sequence in pET22b, C-terminal 10His epitope, ApR	This study
pBB45	substitution of the codon coding His935 in Ala of PA0262 in pBB44, C-terminal 10His epitope, ApR	This study
pBB46	substitution of the codon coding Glu936 in Ala of PA0262 in pBB44, C-terminal 10His epitope, ApR	This study

Table 2: Oligonucleotides used in this study.

Plasmids	Oligonucleotide names and sequences
pBB01	BBO09 : GCAGTGGAACGCCACTGCAGGAACGATAGGGTTCGG
	BBO08 : CCGGGGATCCTCTAGATTAGATTGTCCCCCC
pBB02	BBO09 : GCAGTGGAACGCCACTGCAGGAACGATAGGGTTCGG
	BBO69 : CCGGGGATCCTCTAGATTAAGCAATGCCGCGCCA
pBB03	BBO70 : GCAGTGGAACGCCACTGCAGGGGGCTCCCGGGAAGG
	BBO08 : CCGGGGATCCTCTAGATTAGATTGTCCCCCC
pBB04	BBO12 : GCAGTGGAACGCCACTGCAGGGTTTCCAAACTGCCA
	BBO11 : CCGGGGATCCTCTAGATCATGGCTTCTCTCC
pBB05	BBO15 : GCAGTGGAACGCCACTGCAGGGCCGAGTCGCCGCTA
	BBO14 : CCGGGGATCCTCTAGACTATAAATCGTCCTG
pBB06	BBO15 : GCAGTGGAACGCCACTGCAGGGCCGAGTCGCCGCTA
	BBO67 : CCGGGGATCCTCTAGATTATCGGGGTCGATCCG
pBB07	BBO68 : GCAGTGGAACGCCACTGCAGGCTGGGGCTGGCGCAA
	BBO14 : CCGGGGATCCTCTAGACTATAAATCGTCCTG
pBB08	BBO19 : GCAGTGGAACGCCACTGCAGGGCCAACGACGAGTCC
	BBO17 : CCGGGGATCCTCTAGATCAAACTGCGGAGGCGGG
pBB09	BBO19 : GCAGTGGAACGCCACTGCAGGGCCAACGACGAGTCC
	BBO18 : CCGGGGATCCTCTAGATCAGTATCCCGTTGG
pBB10	BBO07 : GCGCACGCGGCGGGCTGCAGGGAACGATAGGGTTCGG
	BBO08 : CCGGGGATCCTCTAGATTAGATTGTCCCCCC
pBB11	BBO10 :GCGCACGCGGCGGGCTGCAGGGGTTTCCAAACTGCCA
	BBO11 : CCGGGGATCCTCTAGATCATGGCTTCTCTCC
pBB12	BBO12 : GCGCACGCGGCGGGCTGCAGGGGCCGAGTCGCCGCTA
	BBO14 : CCGGGGATCCTCTAGACTATAAATCGTCCTG
pBB13	BBO16 : GCGCACGCGGCGGGCTGCAGGGGCCAACGACGAGTCC
	BBO17 : CCGGGGATCCTCTAGATCAAACTGCGGAGGCGGG
pBB14	BBO16 : GCGCACGCGGCGGGCTGCAGGGGCCAACGACGAGTCC
	BBO18 : CCGGGGATCCTCTAGATCAGTATCCCGTTGG
pBB15	BBO24 : CCAAGCTTGCATGCCTGCAGATGAACGATAGGGTT
	BBO25 : CGGTACCCGGGGATCCTCGATTGTCCCCCCAAA
pBB16	BBO26 : CCAAGCTTGCATGCCTGCAGATGGTTTCCAAACTGCCA
	BBO27 : CGGTACCCGGGGATCCTCTGGCTTCTCTCCTTG
pBB17	BBO28 : CCAAGCTTGCATGCCTGCAGATGGCCGAGTCGCCG
	BBO29 : CGGTACCCGGGGATCCTCTAAATCGTCCTGCCA
pBB18	BBO39 : CCAAGCTTGCATGCCTGCAGATGGCCAACGACGAG
	BBO40 : CGGTACCCGGGGATCCTCAACTGCGGAGGCGG
pBB19	BBO39 : CCAAGCTTGCATGCCTGCAGATGGCCAACGACGAG
	BBO41 : CGGTACCCGGGGATCCTCGTATCCCGTTGGGAA
pBB20	BBO24 : CCAAGCTTGCATGCCTGCAGATGAACGATAGGGTT
	BBO25 : CGGTACCCGGGGATCCTCGATTGTCCCCCCAAA
pBB21	BBO26 : CCAAGCTTGCATGCCTGCAGATGGTTTCCAAACTGCCA
	BBO27 : CGGTACCCGGGGATCCTCTGGCTTCTCTCCTTG
pBB22	BBO28 : CCAAGCTTGCATGCCTGCAGATGGCCGAGTCGCCG
	BBO29 : CGGTACCCGGGGATCCTCTAAATCGTCCTGCCA
pBB23	BBO39 : CCAAGCTTGCATGCCTGCAGATGGCCAACGACGAG
	BBO40 : CGGTACCCGGGGATCCTCAACTGCGGAGGCGG
pBB24	BBO39 : CCAAGCTTGCATGCCTGCAGATGGCCAACGACGAG
	BBO41 : CGGTACCCGGGGATCCTCGTATCCCGTTGGGAA
pBB25	BBO36 : AAGGAGATATACATATGATGCGTCAAAGGGAC
	BBO37 : TTTTTCGAACTGCGGGTGGCTCCAAgcgcTGTATCCCGTTGG
	BBO34bis : GCGTGGCCGGCCGATATCTCATTTTTCGAACTGCGGGTGGCTCCAAGCGCT
pBB26	BBO05 : AAGGAGATATACATATGGTTTCCAAACTGCCA
	BBO34 : TTTTTCGAACTGCGGGTGGCTCCAAgcgcTTGGCTTCTCTCCTTG
	BBO34bis : GCGTGGCCGGCCGATATCTCATTTTTCGAACTGCGGGTGGCTCCAAGCGCT
pBB27	BBO06 : TTTTTCGAACTGCGGGTGGCTCCAAgcgcTTGGCTTCTCTCCTTG
	BBO35 : TTTTTCGAACTGCGGGTGGCTCCAAgcgcTTAAATCGTCCTGCCA
	BBO34bis : GCGTGGCCGGCCGATATCTCATTTTTCGAACTGCGGGTGGCTCCAAGCGCT
pBB28	BBO80 : TTTTTCGAACTGCGGGTGGCTCCAAgcgcTTAAATCGTCCTGCCA
	BBO72 : TCAATGTTAATGATGATGGTGATGGTGATGGTGATGATGTAAATCGTCCTGCCAG
	BBO71bis : CCGCAAGCTTGTCGATCAATGTTAATGATGATGGTGATGGTGATGGTGATGATG

pBB29	VTO26 : CAGGTCGACGGATCCCCGGG GGTTGGAGCCGGGAAAGACGT
	VTO27 : GCATTAGATTGTCCCCCGTTCATGGCTTCTCT
	VTO28 : AGAGAAGCCATGAACGGGGGGACAATCTAATGC
	VTO29 : TATGCATCCGCGGGCCCGGGGAACAACGCCTCCAGTACTCCTTCAGG
pBB30	BBO22 : CAGGTCGACGGATCCCCGGGCCGGCATGGTTCTGGTCACC
	BBO23 : CGCGGGACACCATCGACCTACATTAGATTGTCCCCCCAAA
	BBO24 : TTTGGGGGGACAATCTAATGTAGGTCGATGGTGTCCCG
	VTO35 : TATGCATCCGCGGGCCCGGGAACGGCGCTCTCCCAGGACC
pBB31	VTO32 : CAGGTCGACGGATCCCCGGGCGCCAAGGTCGATATGAAACACACGA
	VTO36 : GCATTAGATTGTCCCCCCATTGGTCAGTATCC
	VTO37 : TACTGACCAATGGGGGGGACAATCTAATGCGCT
	VTO29 : TATGCATCCGCGGGCCCGGGGAACAACGCCTCCAGTACTCCTTCAGG
pBB32	BBO55 : CAGGTCGACGGATCCCCGGGGAAATGCAAGACCTTGCTCA
	BBO56 : GGACGATTTAAGGGTTAGGGATAGGCTTACCTGGCTTCTCT
	BBO57 : CCTATCCCTAACCCTCTCCTCGGTCTCGATTCTACGTGAAAGGAGAGAA
	BBO58 : TATGCATCCGCGGGCCCGGGAGCGAGATGAAACCGAACAG
pBB33	BBO52 : CAGGTCGACGGATCCCCGGGGAAGCTCTCACCGCACAACT
	BBO53 : ATCGAGACCGAGGAGAGGGTTAGGGATAGGCTTACCTAAATCGTCC
	BBO54 : CCTATCCCTAACCCTCTCCTCGGTCTCGATTCTACGTGAGTCGATGGTG
	VTO35 : TATGCATCCGCGGGCCCGGGAACGGCGCTCTCCCAGGACC
pBB34	BBO73 : CAGGTCGACGGATCCCCGGGTGGGCACTTTCAACGACAAG
	BBO74 : ATGGTGATGGTGATGATGCCCTGCCACCGGCGAGCGCC
	BBO75 : CATCACCATCACCATTGAGTTGACAGGCACACCGGTCG
	BBO76 : TATGCATCCGCGGGCCCGGGGTTCCAGCAGCAGTTCGTAG
pBB35	BBO30 : CAGGTCGACGGATCCCCGGGCCGGCATGGTTCTGGTCACC
	SBO157 : TGAGAGCATCAAGCGGATTAGATTGTCCCCCCAA
	SBO158 : TTGGGGGGACAATCTAATCCGCTTGATGCTCTCA
	SBO163 : TATGCATCCGCGGGCCCGGG TGAACAACGCCTCCAGTACT
pBB36	BBO30 : CAGGTCGACGGATCCCCGGGCCGGCATGGTTCTGGTCACC
	SBO159 : ACTGGCAAAGCAGCGGATCGAGGATAGCGGCGA
	SBO160 : TCGCCGCTATCCTCGATCCGCTGCTTTGCCAGT
	SBO163 : TATGCATCCGCGGGCCCGGG TGAACAACGCCTCCAGTACT
pBB37	BBO30 : CAGGTCGACGGATCCCCGGGCCGGCATGGTTCTGGTCACC
	SBO161 : ATAAGCATCAGCGGAGATGGTGGCCTCCTTGGC
	SBO162 : GCCAAGGAGGCCACCATCTCCGCTGATGCTTAT
	SBO163 : TATGCATCCGCGGGCCCGGG TGAACAACGCCTCCAGTACT
pBB38	BBO42 : GCTTGATATCGAATTCGAGGTCGGACTATCC
	BBO46 : TCGGCGTTGGGATACGTTCCTTGTCGTTG
	BBO47 : AACGTATCCCAACGCCGAGGAGCGGCAAG
	BBO48 : CGGGCTGCAGGAATTAGATTGTCCCCCC
pBB39	BBO42 : GCTTGATATCGAATTCGAGGTCGGACTATCC
	BBO43 : GTTTTTCAGGGATACGTTCCTTGTCGTTG
	BBO44 : AACGTATCCCTGAAAAACTTCCCAACGGG
	BBO45 : CGGGCTGCAGGAATTCTCATGGCTTCTCTCC
pBB40	BBO42 : GCTTGATATCGAATTCGAGGTCGGACTATCC
	BBO49 : ACCGCCTGCGGATACGTTCCTTGTCGTTG
	BBO50 : AACGTATCCGCAGGCGGTGCGCTTTGGGG
	BBO51 : CGGGCTGCAGGAACTATAAATCGTCCTG
pBB41	BBO36 : AAGGAGATATACATATGATGCGTCAAAGGGAC
	BBO107 : GCGTGGCCGGCCGATATCTCATTTTTCGAACTGCGGGTGGCTCCAAGCGCTAACTGCGGAGGCGGG
pBB42	BBO36 : AAGGAGATATACATATGATGCGTCAAAGGGAC
	BBO103 : TTTTTCGAACTGCGGGTGGCTCCAAGCGCTGGCCGCGCCGACATG
	BBO37bis : GCGTGGCCGGCCGATATCTCATTTTTCGAACTGCGGGTGGCTCCAAgcgcT
pBB43	BBO93 : AAGGAGATATACATATGATGGAGCACCAGGGCGTG
	BBO94 : GCGTGGCCGGCCGATATCTCAATGTTAATGATGATGGTGATGGTGATGGTGATGATG
pBB44	BBO86 : CCGGCGATGGCCATGGGGGAGCACCAGGGCGTG
	BBO87 : ATGTTAATGATGATGGTGATGGTGATGGTGATGATGGTATCCCGTTGGGAA
	BBO87bis : GGTGGTGGTGCTCGAGTCAATGTTAATGATGATGGTGATGGTGATGGTGATGATG
pBB45	BBO88 : CAGGGTCTGTTCATTGCCGAAATGACCCATGTC
	BBO89 : GACATGGGTCATTTCGGCAATGAACAGACCCTG
pBB46	BBO95 : GGTCTGTTCATTCACGCCATGACCCATGTCTGG
	BBO96 : CCAGACATGGGTCATGGCGTGAATGAACAGACC
pVT1	VTO01 : AGGAGATATACCATGGATGAACGATAGGGTT
	VTO02 : CCGCAAGCTTGTCGACTCAATGTTAATGATGATGGTGATGGTGATGGTGATGATGGATTGTCCCCCCAAA
pVT3	VTO10 : AGGAGATATACCATGGATGAACGATAGGGTT
	VTO11 : CCGCAAGCTTGTCGACTCAATGTTAATGATGATGGTGATGGTGATGGTGATGATGTGTCCCTCCTTT
pVT8	VTO06 : AAGGAGATATACATATGAAATGCAAGACCTTG
	VTO07 : GCGTGGCCGGCCGATATCTCACGTAGAATCGAGACCGAGGAGAGGGTTAGGGATAGGCTTACCTGGCTTCTCTCCTTG
pVT9	VTO08 : AAGGAGATATACATATGCGCTTGATGCTCTCA
	VTO09 : GCGTGGCCGGCCGATATCTCACGTAGAATCGAGACCGAGGAGAGGGTTAGGGATAGGCTTACCTAAATCGTCCTGCCA
pSBC81	VTO51 : CCGGCGATGGCCATGGGGAACGATAGGGTTCGG
	VTO52 : GGTGGTGGTGCTCGAGGATTGTCCCCCCAAA
pSBC107-110	CSO31 : CTCGATTCTACGTGAGATATCTTTAAGAAGGAGATATACATAT
	CSO32 : GCGTGGCCGGCCGATATCTCAGTGGTGGTGGTGGTGGTG

Supplementary Materials and Methods

Fractionation of *E. coli* cells into spheroplasts (cytoplasm and membranes) and periplasmic fractions were done as described previously (Gimenez et al., 2018)

Supplementary References

Battesti, A. and Bouveret, E. (2012) The bacterial two-hybrid system based on adenylate cyclase reconstitution in *Escherichia coli*, *Methods.* 58 325–334.

Gimenez, M.R., Chandra, G., Van Overvelt, P., Voulhoux, R., Bleves, S., and Ize, B. (2018) Genome wide identification and experimental validation of *Pseudomonas aeruginosa* Tat substrates, *Sci Rep.* 8(1):11950

Hoang, T.T., Kutchma, A.J., Becher, A., and Schweizer, H.P. (2000) Integration-proficient plasmids for *Pseudomonas aeruginosa*: site-specific integration and use for engineering of reporter and expression strains. *Plasmid.* 43(1):59-72.

Kaniga, K., Delor, I., and Cornelis, G.R. (1991) A wide-host-range suicide vector for improving reverse genetics in gram-negative bacteria: inactivation of the blaA gene of *Yersinia enterocolitica*, *Gene.* (1991) 109(1):137-41.

Mercy, C., Ize, B., Salcedo, S.P., de Bentzmann, S . and Bigot S. (2016) Functional Characterization of *Pseudomonas* Contact Dependent Growth Inhibition (CDI) Systems. *PLoS One.* 11(1):e0147435

Newman, J.R. and Fuqua, C. (1999) Broad-host-range expression vectors that carry the L-arabinose-inducible *Escherichia coli araBAD* promoter and the *araC* regulator. *Gene.* 227(2):197-203.

Sana, T. G., Berni, B., and Bleves, S. (2016a). The T6SSs of Pseudomonas aeruginosa Strain PAO1 and Their Effectors: Beyond Bacterial-Cell Targeting. *Front Cell Infect Microbiol* 6, 61. doi:10.3389/fcimb.2016.00061.

Sana, T. G., Hachani, A., Bucior, I., Soscia, C., Garvis, S., Termine, E., et al. (2012). The second type VI secretion system of Pseudomonas aeruginosa strain PAO1 is regulated by quorum sensing and Fur and modulates internalization in epithelial cells. *J. Biol. Chem.* 287, 27095–27105. doi:10.1074/jbc.M112.376368.

Supplementary figure 1

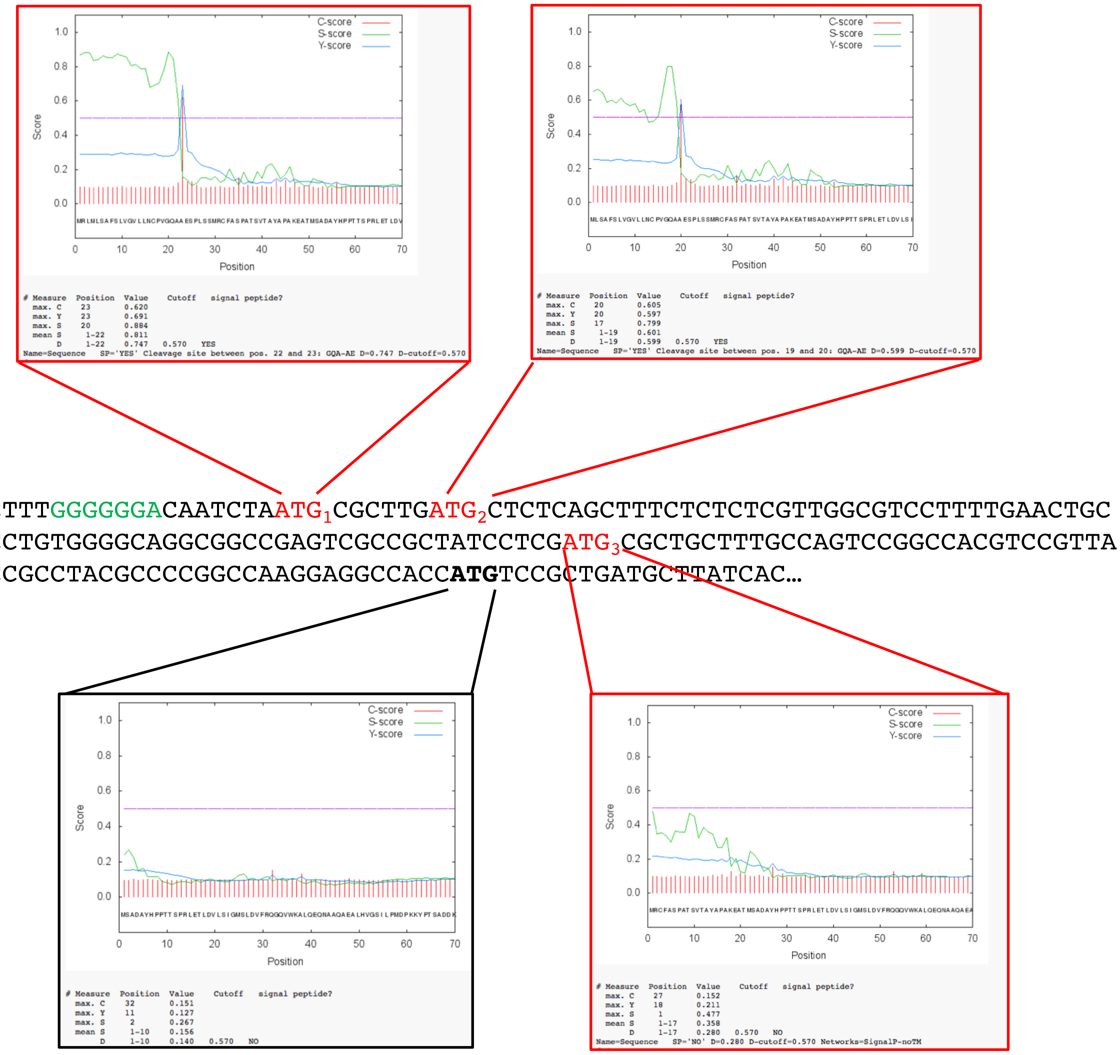

Fig. S1 : *In silico* analysis of the nucleic and protein sequence of Tla3 (PA0259). For the analysis of the PA0259 sequence, three translation initiation codons (in red) upstream of the annotated ATG on the genome (in bold) and in phase with the PA0259 sequence were taken into consideration. The green sequence represents a putative Shine-Dalgarno sequence for the ATG_1 initiation codon. SignalP 4.1 program was used to locate signal sequence at the N-terminus of the fours corresponding proteins. The program predicts the residue where the cleavage site (C-score) takes place, the most probable position of the signal sequence (S-score) and combines these two approaches (Y- and D-scores).

Supplementary Figure 2

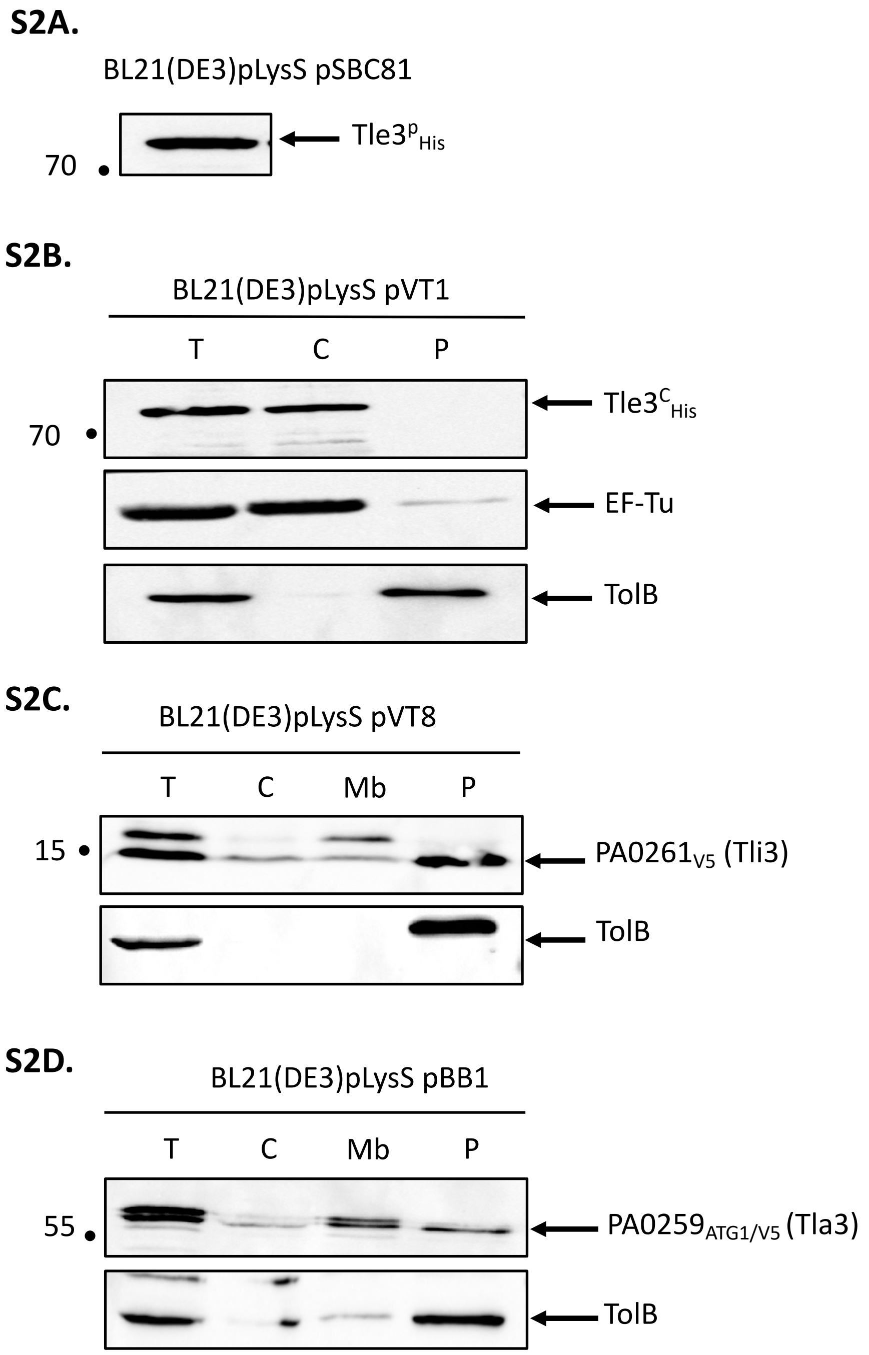

Fig. S2 : Production of Tle3^{p} (S2A) and localization of Tle3^{c}, Til3 and Tla3$_{ATG1}$ in *E. coli* BL21(DE3)pLysS (S2B-D). Except for Tle3^{P} whose production promotes cell lysis, bacteria were subjected to fractionation and immunoblotting using antibodies directed against the His Tag, V5 tag, EF-Tu and TolB. EF-Tu and TolB were used as cytoplasmic and periplasmic controls respectively. T: whole cell, C: cytoplasm, Mb: total membrane, P: periplasm. The position of the proteins and the molecular mass markers (in kDa) are indicated.

Supplementary figure 3

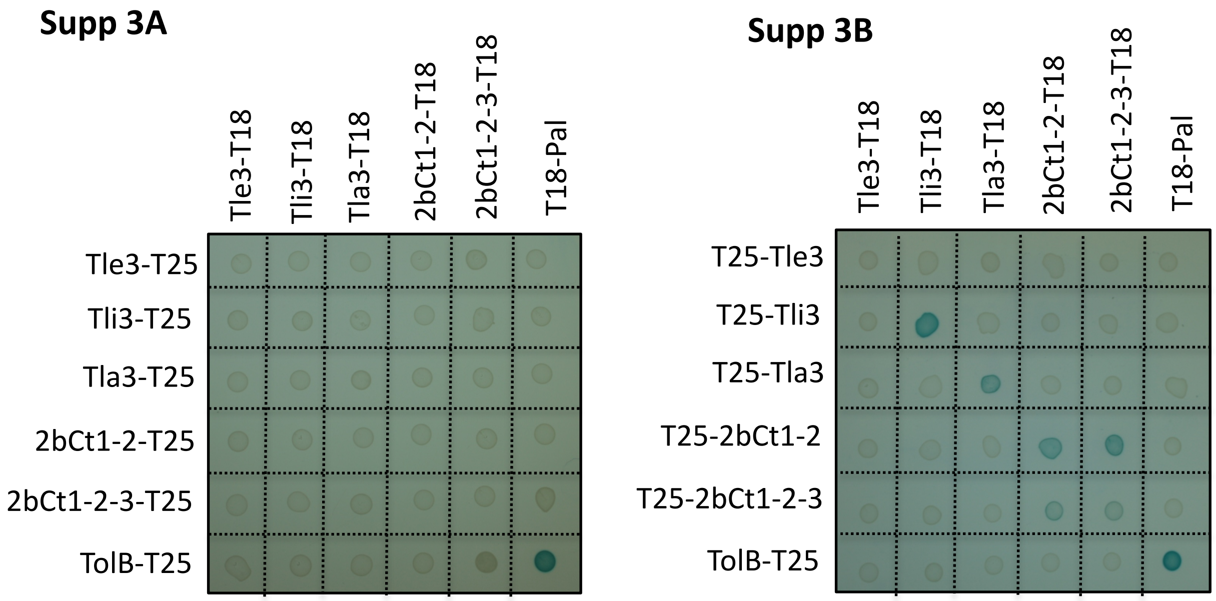

Fig. S3: Bacterial two-hybrid assay (A and B). BTH101 reporter cells producing the indicated proteins or domains fused to the T18 or T25 domain of the *Bordetella* adenylate cyclase were spotted on X-gal indicator plates. The blue color of the colony reflects the interaction between the two proteins. TolB and Pal are two proteins known to interact but unrelated to the T6SS. The experiment was performed in triplicate and a representative result is shown.

Supplementary figure 4

S4A.

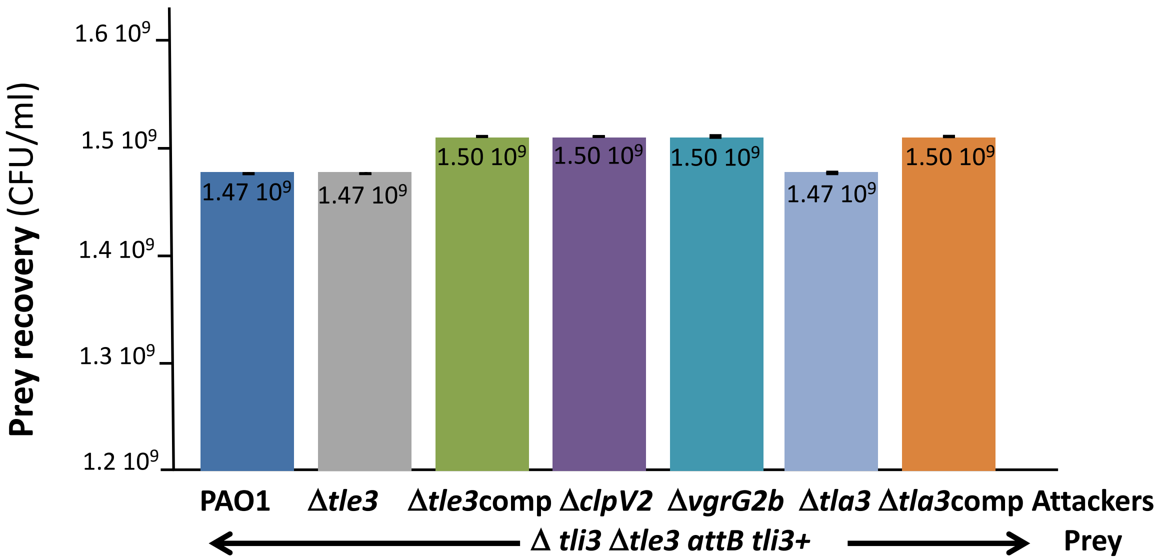

S4B.

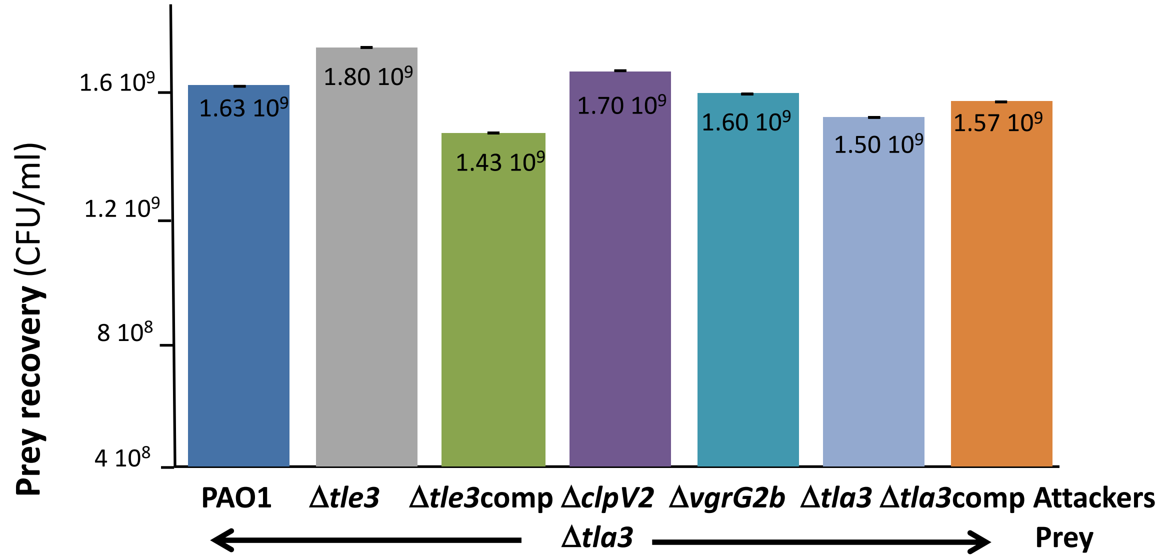

Fig. S4: *P. aeruginosa* growth competition. The *P. aeruginosa* prey strain Δ*tli3*Δ*tle3 attB tli3+* **(S4A)** or Δ*tla3* **(S4B)** was incubated with various *P. aeruginosa* attacker strains as indicated in the figure for 24h at 37°C. The number of recovered prey bacteria is indicated in CFU/ml. "comp" stand for *cis* complementation of the corresponding mutation with a wild-type copy inserted at the *attB* site on *P. aeruginosa* chromosome. Error bars represent ± SEM (n = 3)

Supplementary figure 5

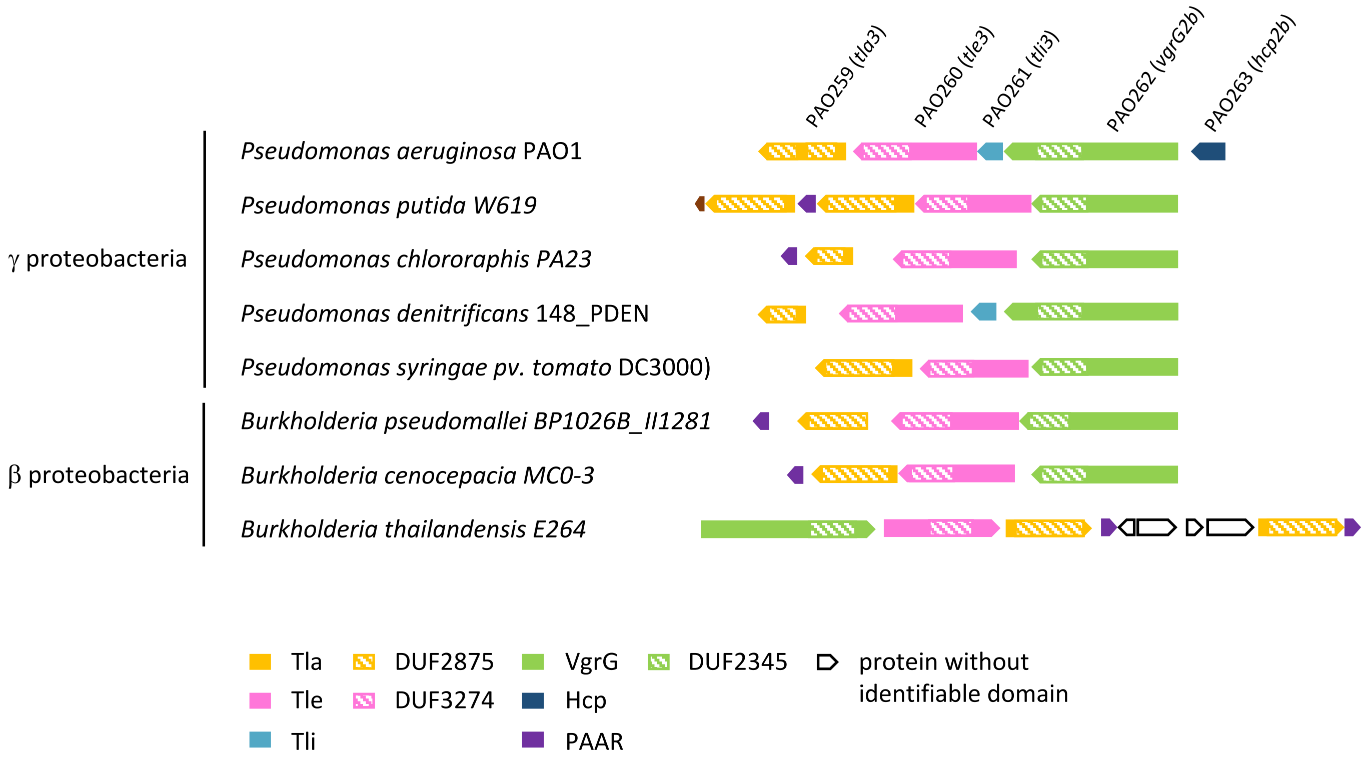

Fig. S5: **Adaptors of Tla3 family DUF2875 are restricted to beta and gamma proteobacteria.**
Tla3 homologues were found in bacteria belonging to γ and β proteobacterial classes. Genetic organization presented at right of each bacterium was deduced from manual inspection of respective bacterial genome and color-coded by the presence of a conserved protein domain as predicted by an NCBI-CD search algorithm. The names of *P. aeruginosa* genes are indicated and the color-coded protein domains are listed and drawn to scale.

Supplementary figure 6

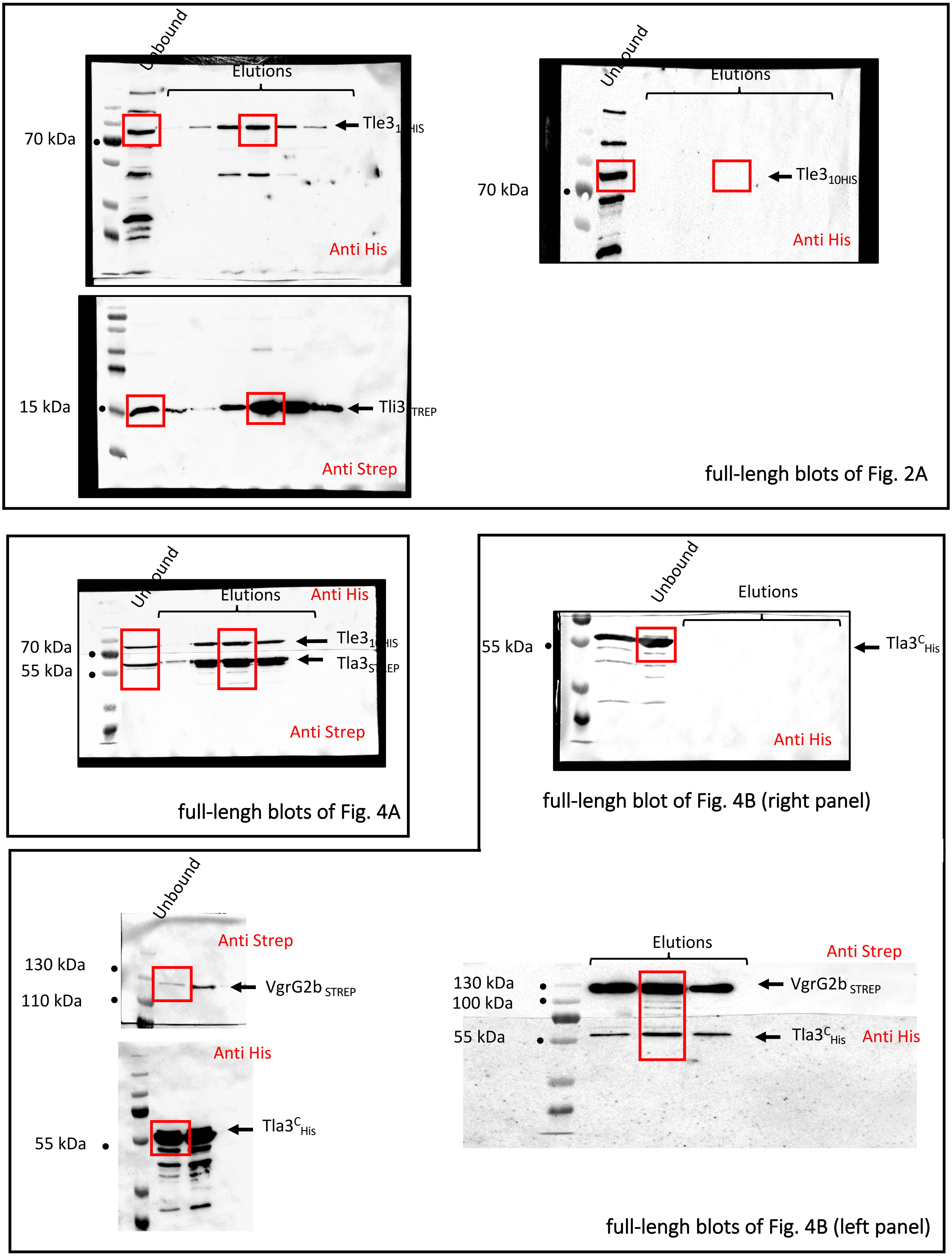

Supplementary figure 6

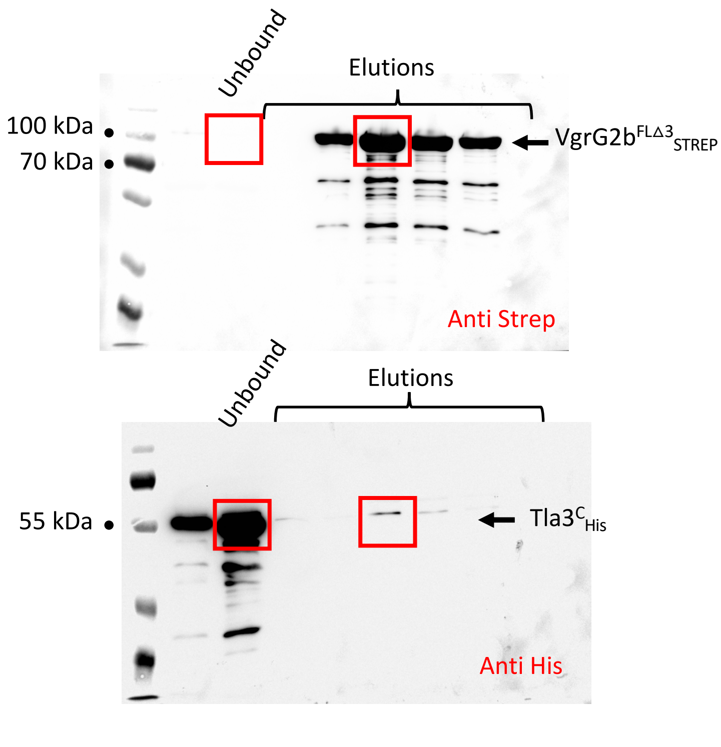

full-lengh blots of Fig. 4C

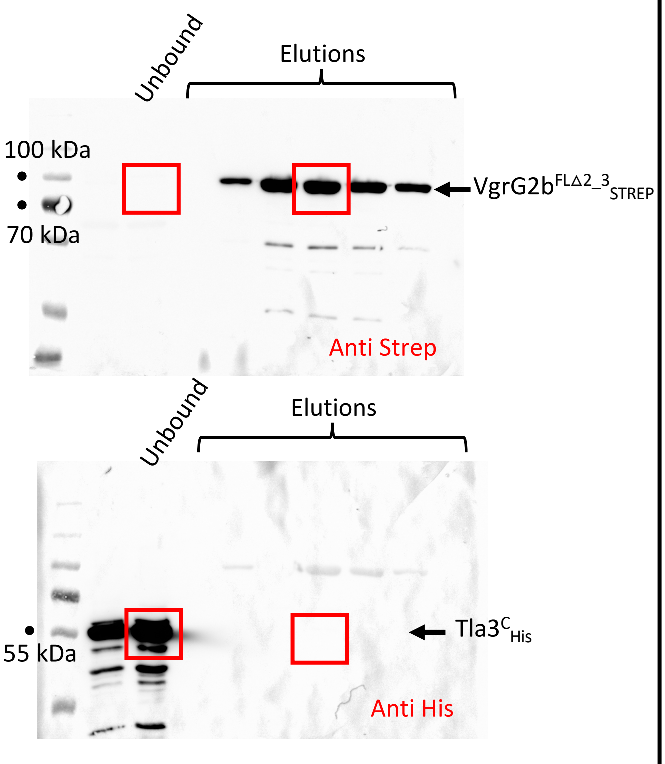

full-lengh blots of Fig. 4D

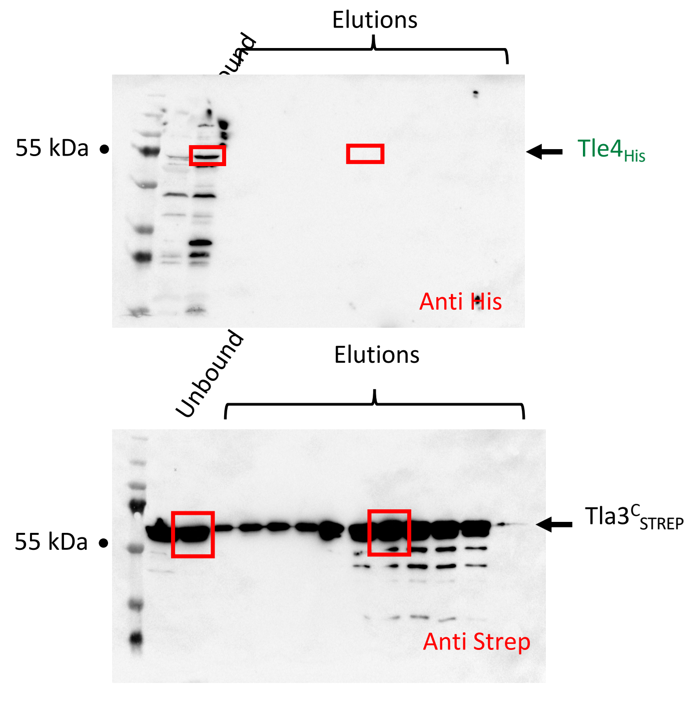

full-lengh blots of Fig. 5D
